# Dialysis withdrawal and symptoms of anxiety and depression: a prospective cohort study

**DOI:** 10.1186/s12882-023-03267-2

**Published:** 2023-07-24

**Authors:** Essam S. El-Magd, Robbert W. Schouten, Els Nadort, Prataap K. Chandie Shaw, Yves F.C. Smets, Louis-Jean Vleming, Friedo W. Dekker, Birit F.P. Broekman, Adriaan Honig, Carl E.H. Siegert

**Affiliations:** 1 Department of Nephrology, OLVG Hospital, Amsterdam, The Netherlands; 2 Department of Internal Medicine, Amsterdam UMC, Amsterdam, The Netherlands; 3 Department of Psychiatry and Medical Psychology, OLVG Hospital, Amsterdam, The Netherlands; 4 Department of Nephrology, HMC Hospital, The Hague, The Netherlands; 5 Department of Nephrology, Haga Teaching Hospital, The Hague, The Netherlands; 6 Department of Clinical Epidemiology, Leiden University Medical Center, Leiden, The Netherlands; 7 Department of Psychiatry, Amsterdam UMC, Vrije Universiteit, Amsterdam, The Netherlands; 8 Amsterdam Mental Health, Amsterdam, The Netherlands

**Keywords:** Dialysis withdrawal, Anxiety, Depression, Mortality, Dialysis, Kidney disease

## Abstract

**Background:**

An important aspect of end-of-life decisions in dialysis patients is elective withdrawal from dialysis therapy. Several studies have shown that clinical factors, such as comorbidity, play a role in dialysis withdrawal. The role of symptoms of anxiety and depression is largely unknown. The.

**Methods:**

A prospective multi-center study has been set up to investigate anxiety and depressive symptoms longitudinally in dialysis patients. Anxiety and depressive symptoms were investigated using the Beck Anxiety Inventory (BAI) and Beck Depression Inventory (BDI) as baseline. Adverse events, including dialysis withdrawal and mortality were registered during follow-up. Multivariable cox proportional hazard models were used with anxiety and depression as the independent variable and dialysis withdrawal as the outcome variable. Models included age, sex, ethnicity and a set of clinical comorbidities.

**Results:**

A total of 687 patients were included between 2012 and 2017, with a median follow-up of 3.2 years. A total of 48 patients (7%) withdrew from dialysis therapy, and subsequently deceased. Anxiety and depressive symptoms at baseline showed an association with dialysis withdrawal with hazard ratios of 2.31 (1.09–4.88) for anxiety and 2.56 (1.27–5.15) for depressive symptoms, independent of somatic comorbidities.

**Discussion:**

Withdrawal from dialysis therapy is associated with anxiety and depressive symptoms. Dialysis patients with more severe depressive and anxiety symptoms were more vulnerable for dialysis withdrawal. Insight in factors that play a role in dialysis withdrawal could aid patients and clinicians making an informed decision and develop clinical guidelines.

**Supplementary Information:**

The online version contains supplementary material available at 10.1186/s12882-023-03267-2.

## Background

Dialysis withdrawal has become a more common occurrence on the dialysis wards. Recent data shows that withdrawal rates have doubled in the last decade [1–3]. Estimated incidence rate range from 7 to 20% [4, 5]. The variation is partly explained due to a high degree of heterogeneity in the definition of withdrawal from dialysis [6–8]. A recent study identified age, sex, recently starting dialysis and somatic comorbidities as potential risk factors for dialysis withdrawal. The authors do note that psychiatric comorbidities, such as depression, could be associated with dialysis withdrawal, but that no psychiatric data was collected [10].

Anxiety and depression are the most common psychiatric comorbidities among dialysis patients with an estimated prevalence of 15–38% for anxiety and 37–42% for depressive symptoms. Data on the role of psychiatric factors influencing dialysis withdrawal is scarce [9, 10]. Only one smaller study in 2006 with 202, mostly Caucasian (90%), dialysis patients has investigated the association between depression and dialysis withdrawal, which showed an increased risk of withdrawal when patients reported depressive symptoms [5]. Another study by Lacson et al., which used the mental component scores of the SF-36 as a marker for depression found a similar association [11]. To the best of our knowledge there has been no data on the association between anxiety symptoms and withdrawal.

Currently it is unknown how to handle symptoms of depression and anxiety in relation to end-of-life care options in end-stage renal disease patients [4, 12]. Identification of factors influencing dialysis continuation or withdrawal could aid in making informed decisions to withdraw from dialysis therapy.

The primary aims of this study are to investigate the association between: (1) depressive symptoms and dialysis withdrawal, and (2) anxiety symptoms and dialysis withdrawal. Secondary aims are to investigate the relationship between mental and physical component scores of Quality of Life (QoL) questionnaires and dialysis withdrawal.

## Methods

### Study cohort and follow-up

Data was collected from the observational prospective cohort study Depression-related factors and outcomes In dialysis patients with Various Ethnicities and Races Study.

(DIVERS) [10, 13–17]. The study cohort consisted of prevalent and incident dialysis patients from 10 dialysis centers in the Netherlands. These include 2 centers of the OLVG hospital, 2 centers of the HMC hospital, 3 centers of the Haga hospital and 3 centers of the VUmc university hospital. Patients were included between June 2012 and October 2016. All patients who met the inclusion criteria were approached for study participation during dialysis treatment or during an outpatient appointment for patients receiving peritoneal dialysis therapy. Inclusion criteria were being at least 18 years of age and having a dialysis vintage of at least 90 days. Patients who were unable to fill in self-reported questionnaires were excluded. To improve generalizability, all questionnaires and variables were available in Dutch, English, Turkish, and Moroccan Arabic translations, furthermore patients with disabilities or illiteracy which impaired them to fill in the questionnaires, were helped by a trained research assistant. Before inclusion, all patients gave informed consent. Legal guardians signed informed consent for participants who were unable to sign the informed consent themselves, either due to cognitive disabilities or illiteracy. Patients who receive renal replacement therapy in the Netherlands are fully covered by an obligatory healthcare insurance. Patients on dialysis therapy are fully covered by all public healthcare insurance policies [18].This study was approved by the medical ethics committees of all participating hospitals and was carried out in accordance with the Declaration of Helsinki.

### Demographic and clinical data

At baseline, the following sociodemographic and clinical data were collected from electronic medical records: age, sex, dialysis modality and vintage, comorbid conditions, transplant waiting list status and current medication use. Incident patients were defined as new patients on renal replacement therapy for more than 90 days and less than 180 days. The primary cause of kidney disease was classified according to the European Renal Association–European Dialysis and Transplant Association (ERA-EDTA) coding system and divided into 4 groups (diabetes mellitus, glomerulonephritis, renal vascular disease, and other) [19]. The level of comorbidity was defined according to the Davies comorbidity index, indicating no, intermediate or severe comorbidity, this 3-point severity index was used in the multivariable analyses [20]. We collected the following characteristics through self-reported questionnaires: immigrant status (defined as immigrant status based on the country of birth) [21], marital status, number of children, educational level, religion, employment status, current smoking and alcohol use, and previous depression.

### Depressive and anxiety symptoms and quality of life

Baseline depressive and anxiety symptoms were measured using-self-report questionnaires, The Beck Depression Inventory second edition (BDI-II) and Beck Anxiety Inventory (BAI), respectively [22, 23]. Both questionnaires consist out of 21 questions relating to cognitive and somatic symptoms of depression and anxiety. Respondents were asked to rate the severity of each of these symptoms in the past week on a scale ranging from 0 to 3; not at all to severely burdened. A total score was calculated by summing all items, with a minimum of 0 and maximum of 63.

Both the BDI and BAI have been validated in a large variety of cohorts of patients with various anxiety and depressive disorders diagnosed with the Structured Clinical Interview for Diagnostic and Statistical Manual of Mental Disorders, 4th. Edition (SCID-1), including cohorts of patients with other chronic somatic diseases. This study used validated BAI translations in Dutch, English, Turkish and Moroccan Arabic. The BDI-II and BAI both have high internal consistency, respectively a Cronbach α of 0.91 and a Cronbach α of 0.92. The BDI-II has a high one-week test-retest reliability of 093 and the BAI has a reliability of 0.75 [22, 24–26].

The presence of depressive and anxiety symptoms was also dichotomized by using cut-off values. For the BDI, a cut-off value of ≥ 13 was used as this cut-off value has been validated in a Dutch cohort of dialysis patients [27]. For the BAI, the cut-off of ≥ 16 was used in the analyses was based on the manual provided by Beck and Steer indicating “clinically significant” anxiety symptoms. Despite the frequent use of the BAI in patient groups with somatic diseases, this cut-off value for the BAI has not yet been validated in dialysis patients. In this study the term ‘anxiety’ and ‘depression’ refers to patients who scored above the predefined cut-off scores for clinically relevant symptoms, not to a clinical diagnosis based on the DSM-5. Furthermore, patients who answered the question on the BDI related to suicidal ideation were screened afterwards for active suicidal thoughts or plans. Patients who were deemed to have a heightened risk for suicide were excluded from the study and their primary physician were informed to facilitate further treatment or aid.

Quality of Life (QoL) was measured using the 12-Item Short Form Health Survey (SF-12), which includes a Mental component score (MCS) and a Physical component score (PCS) [28]. The medical outcome survey Short Form 12 (SF-12) has been widely used and validated as a quality of life (QoL) assessment tool in dialysis patients [29]. Furthermore, studies have shown that the SF-12 is associated with all-cause mortality in chronic dialysis patients [29]. To the best of our knowledge, no cut-off score for dialysis patients has been validated. To dichotomize the result the median value of the MCS and PCS will be used as cut-off value to compare patients below and above the median value. Both the continuous score of the MCS and PCS subscales of SF-12 and the dichotomized variables of the SF-12 MCS and PCS subscales will be used in the analyses.

### Assessment of outcome: dialysis withdrawal

Mortality and withdrawal rates were extracted from medical records. Cause of death was first classified according to the European Renal Association-European Dialysis and Transplant Association (ERA-EDTA) coding system [19]. All mortalities were then evaluated by examination of medical records and physician notes. Dialysis withdrawal was defined as a patient preference to acutely stop with dialysis therapy without an immediate medical reason or indication to stop the dialysis therapy. This medical indication was judged and coded by the treating physician. If the records were not clear, the primary physician was interviewed.

### Statistical analysis

All statistical analyses were performed using either SPSS for Windows, version 24 (IBM Corp), and R-studio version 3.5.3. Baseline characteristics were stratified by the presence or absence of symptoms of depression or anxiety, defined as patients scoring above or below the predefined cutoff scores of ≥ 13 and ≥ 16 respectively. Incidence rates of dialysis withdrawal will be calculated using events per 1000-person year using follow-up time in days after inclusion in the DIVERS study and number of events.

Cox proportional hazard regression analysis was used to calculate crude Hazard Ratio’s (HR) to determine the relative risk of anxiety and/or depression on dialysis withdrawal. Multivariate adjustment of the crude HR’s was performed to adjust for possible confounders using the following variables; age, sex, ethnicity and comorbidity using the Davies comorbidity score in 3 severity categories. An a priori sequential order in the regression model was used to examine the effect of these possible confounders in the following steps:

1. Crude, univariable exposure (depression or anxiety or both).
2. Adding age, sex and ethnicity to the model.
3. Adding comorbidity to model 2.

Primary analyses will focus on model 2 and model 3. All regression models will be tested separately for anxiety symptoms and depressive symptoms and for the combination of both anxiety and depression.

Secondary analyses include analyzing the continuous BDI and BAI scores, the continuous MCS and PCS scores of the SF-12 QoL and the dichotomized MCS and PCS variable of the SF-12 QoL and their association with dialysis withdrawal using the same multivariate model described above. Furthermore, to assess the impact of missing data on results BDI and BAI scores were imputed using multiple imputation (10 repetitions.)

Lastly, patients’ characteristics will be summarized using a stratification on the outcome: dialysis withdrawal. This table will be used to provide insight in patients’ characteristics that might be associated with dialysis withdrawal. This step will be viewed as explorative without formal tests, which could aid in raising new research questions.

### Sensitivity analyses

A sensitivity analysis excluding patients who received a kidney transplant during follow-up will also be performed. Dialysis populations are extremely heterogenous, with certain patients having no hope of ever receiving a transplant due to health reasons or otherwise compared to others who still have an option for transplant. Patients with no other option than dialysis are faced with the fact that they have to spend the rest of their lives on dialysis. Faced with these reality patients are likely more prone to develop reactive depression to their situation. This sensitivity analysis will attempt to correct for this by excluding patients who received a kidney transplant.

## Results

### Baseline characteristics

Baseline characteristics of the total cohort of 687 patients are shown in Table 1. The mean age was 65 ± 15 years, and 62% of the patients were male. The cohort was multi-ethnic and multi-religious, with 300 (48%) participants having an immigration background, 99 (17%) participants being a Muslim, 213 (36%) Christian and 44 (7%) Hindu. Most patients were on hemodialysis (88%), with 203 (30%) of the patients being on the waiting list for kidney transplantation. Both incident (37%) and prevalent (63%) patients were included, with an average dialysis vintage for prevalent patients of 13 months [IQR: 4–47]. The maximum follow-up was 4 years, with a median follow-up of 3.1 years. Patients with depressive symptoms above the cut-off value had an average BDI score of 21.6 ± 8.1 and an average BAI score of 16.3 ± 10.9 and those without clinically significant symptoms had averages BDI scores of 6.4 ± 3.4 and BAI scores of 5.6 ± 6.0. Patients with anxiety symptoms above the cut-off value also had higher average BAI scores of 25.4 ± 9.5 and BDI scores of 22.7 ± 10.4 compared to those with anxiety symptoms below the cut-off, who had average scores of 6.0 ± 4.5 and 9.9 ± 7.4 for BAI and BDI respectively. Suicidal thoughts were present in 36 patients (11%), measured using item 9 on the BDI.

**Table 1:** Baseline characteristics of the Divers cohort

		Anxiety^a^		Depression^b^	
	All	No	Yes	No	Yes
	*n = 687*	*n = 395*	*n = 113*	*n = 305*	*n = 228*
Demographic					
Age (years)	64 ± 15	65 ± 15	62 ± 14	65 ± 16	64 ± 14
Male	424 (62%)	249 (63%)	71 (63%)	208 (68%)	143 (63%)
Ethnicity (% immigrant) . European . Sub-Saharan . North-Africa/Western Asia . South/South-East. Asia . South America/Caribbean	300 (47.8%) 366 (58%) 22 (4%) 54 (9%) 57 (9%) 131 (21%)	163 (43%) 244 (64%) 13 (3%) 19 (5%) 33 (9%) 75 (20%)	67 (61%) 45 (41%) 2 (2%) 17 (16%) 14 (13%) 32 (29%)	124 (40.7%) 193 (65%) 15 (5%) 17 (6%) 24 (8%) 50 (17%)	123 (56%) 109 (49%) 5 (2%) 21 (10%) 24 (11%) 62 (28%)
Social					
Married	316 (52%)	207 (52%)	55 (49%)	172 (57%)	114 (50%)
Has children	474 (78%)	306 (78%)	87 (78%)	233 (77%)	179 (79%)
Low formal education	332 (56.7%)	201 (51%)	70 (64%)	151 (51%)	133 (60%)
Religion . None . Christian . Islamic . Hinduism . Other	220 (37%) 213 (36%) 99 (17%) 44 (7%) 21 (4%)	159 (41%) 144 (37%) 48 (12%) 24 (6%) 13 (3%)	32 (29%) 32 (29%) 33 (30%) 12 (11%) 3 (3%)	123 (41%) 115 (38%) 38 (13%) 18 (6%) 7 (2%)	76 (34%) 75 (33%) 46 (20%) 21 (9%) 8 (4%)
Not employed	534 (89%)	336 (86%)	107 (95%)	258 (85%)	207 (91%)
Renal and Dialysis					
Incident Patient	240 (36%)	154 (39%)	34 (30%)	122 (40%)	77 (34%)
Dialysis vintage (months)	13 [4–47]	11 [4–45]	28 [5–57]	8 [4–39]	15 [4–48]
Treatment modality: . Hemodialysis . Peritoneal dialysis	601 (88%) 84 (12%)	344 (87%) 51 (13%)	101 (89%) 12 (11%)	274 (89%) 34 (11%)	198 (87%) 30 (13%)
Primary kidney disease: . Diabetic Nephropathy . Renal vascular disease . Glomerulonephritis . Other	155 (24%) 163 (26%) 70 (11%) 247 (39%)	82 (23%) 100 (28%) 40 (10%) 140 (39%)	38 (36%) 18 (17%) 11 (10%) 40 (37%)	56 (20%) 71 (26%) 35 (13%) 115 (42%)	65 (30%) 50 (23%) 22 (10%) 79 (37%)
On waiting list for Tx: . Yes . No, for medical reasons . No, by patient preference	203 (30%) 436 (64%) 46 (7%)	126 (32%) 245 (62%) 24 (6%)	30 (27%) 72 (64%) 11 (10%)	104 (34%) 179 (59%) 22 (7%)	69 (30%) 148 (65%) 11 (5%)
Clinical					
Current smoking	108 (18%)	68 (18%)	23 (21%)	53 (18%)	48 (21%)
Current alcohol use	161 (27%)	110 (28%)	27 (24%)	94 (31%)	53 (24%)
Davies comorbidity score: . None . Intermediate . Severe	183 (27%) 370 (55%) 119 (18%)	109 (29%) 212 (55%) 62 (16%)	24 (21%) 58 (52%) 30 (27%)	90 (31%) 156 (53%) 49 (17%)	51 (23%) 133 (59%) 43 (19%)
Diabetes mellitus	288 (42%)	158 (40%)	58 (52%)	117 (38%)	123 (54%)
Ischemic heart disease	190 (28%)	97 (25%)	41 (36.4%)	77 (26%)	71 (31%)
Peripheral vascular disease	168 (25%)	99 (26%)	27 (24%)	65 (22%)	60 (26%)
Mental health:					
Previous depression	27 (4%)	12 (3%)	6 (5%)	9 (3%)	12 (5%)
Antidepressant use	65 (10%)	28 (7%)	15 (13%)	22 (7%)	28 (12%)
Depression: . BDI Score . BDI ≥ 13 . Suicidal thoughts (item 9)^*^	12.9 ± 9.6 228 (33%) 64 (11%)	9.9 ± 7.4 110 (30%) 30 (8%)	22.7 ± 10.4 80 (83%) 23 (21%)	6.4 ± 3.4 - 6 (2%)	21.6 ± 8.1 - 51 (22%)
Anxiety: . BAI Score . BAI ≥ 16	10.3 ± 10.1 113 (22%)	6.0 ± 4.5 -	25.4 ± 9.5 -	5.6 ± 6.0 16 (6%)	16.3 ± 10.9 80 (42%)
Quality of Life: . Physical component score . Mental component score	38.1 ± 11.1 48.9 ± 10.8	38.2 ± 10.5 50.9 ± 9.5	33.0 ± 10.1 40.0 ± 11.3	40.3 ± 10.4 52.0 ± 8.5	32.9 ± 9.4 41.8 ± 11.0

Values are presented as mean +/- SD, median [IQR] or frequency (percentage).

^a^ The presence of depression is defined as a BDI score ≥ 13

^b^ The presence of anxiety is defined as a BAI score ≥ 16

^*^ Using a grouping variable based on item 9 of the BDI: When patients scored a 1 or higher, they were dichotomized into the group with suicidal thoughts.

### Incidence of withdrawal

A total of 48 patients (7%) withdrew from dialysis therapy during the follow-up of this study, and subsequently passed away. Supplementary table S1 shows the incidence rates of dialysis withdrawal per 1000 person years (py), stratified by the presence of anxiety and depression. Patients who scored above the cut-off value for depressive symptoms showed a withdrawal rate of 40.4/1000 py compared to 27.0/1000 py for patients below the cut-off value, which results in an absolute risk increase of 13.4 withdrawals / 1000 py. For anxiety symptoms, the increase was 6.3 withdrawals / 1000 py. These crude results, without adjusting for confounding, indicated that there is an increase in incidence rate of withdrawal with symptoms of anxiety and/or depression above the clinical cut-off.

### Association between anxiety, depression and dialysis withdrawal

**Fig. 1:**
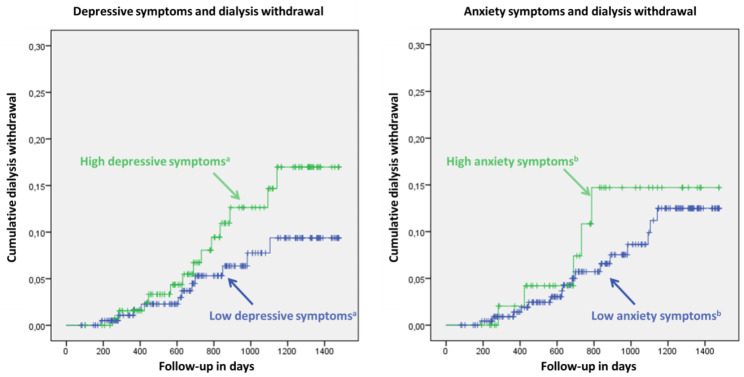
Kaplan Meier curves on cumulative dialysis withdrawal stratified by the presence of anxiety and depressive symptoms ^a^The cut-off value for depressive symptoms was BDI ≥ 13. ^b^The cut-off value for anxiety symptoms was BAI ≥ 16.

Kaplan Meier plots showed an increased risk of dialysis withdrawal in patients who scored above the cut-off value for depression and anxiety, as shown in Fig. 1. The primary analyses using cox proportional hazard regression models are shown in Table 2. The primary analyses included two models; model 1, which includes age, gender and ethnicity and model 2, which additionally includes the Davies comorbidity score besides the variables from model 1. Patients with depressive symptoms had a 2-fold increase in risk of dialysis withdrawal during follow-up, with a Hazard Ratio (HR) of 2.56 (CI: 1.27–5.15, p = 0.009, model 1 in Table 2). When comorbidities were included in the model, the HR did not show a major change with a HR of 2.44 (CI: 1.21–4.93, p = 0.0013, Model 2 in Table 2). When accounting with multiple imputation patients with depressive symptoms still had around a 2-fold increase in risk of dialysis withdrawal when fully corrected, with a HR of 1.95 (1.04–3.77, p = 0.046, Model 2 in supplementary Table S4a).

**Table 2:** Association between depression, anxiety and dialysis withdrawal

*Hazard ratio’s for dialysis withdrawal*	Crude	Model 1 (+ age, gender, ethnicity)	Model 2 (+ comorbidity)
Depressive symptoms^a^	1.64 (0.83–3.25) p = 0.156	2.56 (1.27–5.15) p = 0.009	2.44 (1.21–4.93) p = 0.013
Anxiety symptoms^b^	1.35 (0.66–2.79) p = 0.411	2.31 (1.09–4.88) p = 0.028	2.02 (0.94–4.32) p = 0.071
Both depression and anxiety symptoms^c^	1.95 (0.87–4.38) p = 0.106	3.10 (1.35–7.11) p = 0.008	2.45 (1.05–5.71) p = 0.037

Model 1 includes both the exposure + age, gender and ethnicity

Model 2 includes both the variables from model 1 + additionally the DAVIES comorbidity score, which includes points for ischemic heart disease, heart failure, peripheral vascular disease, malignancy, diabetes, collagen vascular disease, COPD and others

^a^ The presence of depression is defined as a BDI score ≥ 13

^b^ The presence of anxiety is defined as a BAI score ≥ 16

^c^ Based on a grouping variable with both the BDI ≥ 13 AND BAI ≥ 16 versus patients with only anxiety or only depression or none

For anxiety symptoms a similar trend was seen, with an increased risk for dialysis withdrawal for patients who scores above the cut-off value, (HR 2.31 (CI: 1.09–4.88, p = 0.028, model 1 in Table 2)). After the inclusion of comorbidities, the HR and corresponding 95% confidence interval slightly decreased, with a HR of 2.02 (CI: 0.94–4.32, p = 0.071, Model 2 in Table 2). When accounting for missing with multiple imputation the HR decreased to 1.65 (CI: 0.73–3.73, p = 0.226, Model 2 in supplementary Table S4a). Patients with both anxiety and depressive symptoms above the cut-off (BDI ≥ 13 and BAI ≥ 16) had a slight increase in HR compared to the HR from depression and anxiety alone, with a HR of 2.45 in the fully adjusted model (CI: 1.05–5.71, p = 0.037, model 2 in Table 3). Similar to when looking at depression and anxiety alone, accounting for missing with multiple imputation decreased the HR to 1.99 (CI: 1.25–3.17, p = 0.004, Model 2 in supplementary Table S4a). When this group is compared with patients with less symptoms of depression or anxiety, the HR was 3.82 (CI:1.48–9.86, p = 0.006, data not in table). Overall, these results suggest a 2-fold increase in risk for dialysis withdrawal in patients with anxiety and/or depressive symptoms.

**Table 3:** Association between quality of life and dialysis withdrawal

*Hazard ratios for dialysis withdrawal*	Crude	Model 1 (+ age, gender, ethnicity)	Model 2 (+ comorbidity)
Mental component score SF-12 < 50.0*	1.41 (0.78–2.53) p = 0.254	1.91 (1.05–3.49) p = 0.034	1.81 (0.99–3.30) p = 0.053
Physical component score SF-12 < 37.0*	1.61 (0.89–2.91) p = 0.116	1.51 (0.83–2.74) p = 0.178	1.26 (0.69–2.30) p = 0.463

Model 1 includes both the exposure + age, gender and ethnicity

Model 2 includes both the variables from model 1 + additionally the DAVIES comorbidity score, which includes points for ischemic heart disease, heart failure, peripheral vascular disease, malignancy, diabetes, collagen vascular disease, COPD and others

^*^ The higher the score on the SF-12, the better the Quality of Life. A cut-off of 50 was used for the MCS and 37 for the PCS.

### Association between quality of life and dialysis withdrawal

The associations between the QoL component scores and dialysis withdrawal are shown in Table 3 and Supplementary Table 5b. The mental component score showed a HR of 1.91 for dialysis withdrawal (CI:1.05–3.49, p = 0.034, Model 1 in Table 3), where the physical component score showed a non-significant HR of 1.51 (CI: 0.83–2.74, p = 0.178, Model 1 in Table 3). To investigate the effect of the physical quality of life score on the association between depression/anxiety and withdrawal we did an additional analysis using Model 3, in which we added the physical component score as a covariate. Results from this analysis indicated no major differences in the associations between depression/anxiety and withdrawal with a HR of 2.2 (1.04–4.67, p = 0.040) for depression and a HR of 1.95 (0.91–4.17, p = 0.087) for anxiety. These results indicate that the association between anxiety, depression and dialysis withdrawal is largely independent from the physical component of the QoL score.

### Characteristics of patients who withdrew from dialysis therapy

Characteristics of patients who withdrew from dialysis therapy are described in Table 4. Compared to the total cohort, patients who withdrew from dialysis therapy had a longer dialysis vintage, older age, higher education level and slightly more comorbidities. Of the patients who withdrew, 98% were not on the waiting list for transplantation, 55% scored high on the depression scale and 26% on the anxiety scale. The QoL scores did not show major differences compared to other patients.

**Table 4:** Baseline characteristics stratified by dialysis withdrawal, death to other cause and no withdrawal

*Baseline characteristics*	Withdrawal n = 48	Death by other cause^*^ n = 122	No withdrawal^**^ n = 621
Age (years)	77 ± 11	71 ± 11	63 ± 15
Male	30 (63%)	77 (63%)	382 (61%)
Ethnicity (% immigrant) . European . Sub-Saharan . Northern Africa/Western Asia . Southern Asia/South Eastern Asia . South America/Caribbean	10 (22%) 40 (87%) 0 (0%) 0 (0%) 4 (9%) 2 (4%)	45 (40%) 75 (67%) 0 (0%) 5 (5%) 10 (9%) 22 (20%)	281 (50%) 320 (56%) 22 (4%) 51 (9%) 51 (9%) 126 (22%)
Married	22 (52%)	55 (53%)	290 (53%)
Has children	35 (83%)	92 (88%)	432 (79%)
Low education	211 (38%)	45 (48%)	23 (55%)
Employed	0 (0%)	8 (8%)	64 (12%)
Dialysis vintage (months)	33 [6–53]	21 [5–52]	12 [4–45]
Peritoneal Dialysis	6 (13%)	10 (8%)	74 (12%)
Central venous catheter	8 (17%)	13 (11%)	81 (13%)
Residual diuresis > 100 ml/24 h	33 (69%)	79 (65%)	439 (71%)
On waiting list for Tx	1 (2%)	13 (11%)	200 (32%)
*DAVIES comorbidity scale*: . No . Intermediate . Severe	6 (13%) 24 (50%) 18 (38%)	19 (16%) 67 (55%) 36 (30%)	176 (28%) 345 (56%) 100 (16%)
*Comorbidities*: . Diabetes mellitus . Heart failure . Peripheral vascular disease	25 (52%) 14 (29%) 15 (31%)	67 (55%) 27 (22%) 27 (18%)	259 (42%) 96 (16%) 69 (11%)
*Mental health*: . Depression (BDI ≥ 13) . BDI score . Anxiety (BAI ≥ 16) . BAI score . Mental score SF-12 . Physical score SF-12	18 (55%) 14 ± 7 10 (26%) 12 ± 8 49 ± 10 35 ± 11	38 (43%) 14 ± 11 23 (30%) 13 ± 12 48 ± 12 36 ± 12	207 (43%) 13 ± 10 100 (22%) 10 ± 10 49 ± 11 38 ± 11

* The ‘Death by other cause’ group is mutually exclusive with the ‘Withdrawal’ group

** The ‘No withdrawal’ group is the total cohort minus the withdrawal group, this includes patients who died by other causes, who underwent a transplantation or were otherwise censored in the cox models

Almost all patients who withdrew from dialysis therapy were native patients from European origin. Interestingly, none of the 99 Muslim patients withdrew from dialysis, as shown in Supplementary table S2, while 5–7% of the Christian or Hindu patients withdrew from dialysis therapy.

### Sensitivity analyses

Sensitivity analysis performed on the complete case dataset without patients who received transplant shows similar data to our primary analysis, as shown in Supplementary table S4b. A total of n = 28 patients received a transplant during follow-up and were excluded from the sensitivity analysis.

Furthermore, the observational data in Table 4 suggest that education level and dialysis vintage are more strongly associated with dialysis withdrawal than sex. Since our statistical analysis plan was established a priori, we did not change our main analyses, instead we performed 2 additional sensitivity analyses which included education level or dialysis vintage as a covariate instead of sex. These models did not show major differences compared to the original intended models, as shown in Supplementary table S3a and S3b Furthermore,

## Discussion

The aim of this study was to investigate if anxiety and depressive symptoms increased the risk of dialysis withdrawal in End-Stage Kidney Disease (ESKD) patients. During the follow-up of the included 687 dialysis patients, 48 patients decided to electively withdraw from dialysis therapy. High anxiety and depressive symptoms were associated with dialysis withdrawal with hazard ratios of 2.31 (1.09–4.88) for anxiety, 2.56 (1.27–5.15) for depression and 2.45 (1.05–5.71) for concurrent anxiety and depressive symptoms in a multivariable model including age, gender and ethnicity. When somatic comorbidity was included in these models the hazard ratios showed only minor changes, indicating that the effect of anxiety and depressive symptoms on dialysis withdrawal is independent of somatic comorbidities. Likewise, especially the MCS of the SF-12 compared to the PCS of the SF-12 QoL questionnaire increased the risk on dialysis withdrawal with an HR of 1.91 (CI: 1.05–3.49) and HR 1.51 (CI: 0.83–2.74), respectively. Furthermore, these symptoms of depression and anxiety were found to be stable over time and did not show a large variation, as described in another paper with a mixed-model analyses which showed no significant changes in the BDI and BAI scores between the 6-month-interval time points in the same cohort as described in this paper [30].

Current literature shows varying incidence rates of dialysis withdrawal, possibly due to heterogeneous study design and definitions of withdrawal [6–8]. There are no studies on the effect of anxiety on dialysis withdrawal. Only one study investigated the association between depressive symptoms and dialysis withdrawal using a validated questionnaire. While similar to this study being a prospective observational study with self-report questionnaires the study had a comparatively small sample size with 202 patients. This study by McDade et al. found a 1-point increase in the total BDI score was associated with a 5.2% increase in risk of withdrawing [5]. This is in concordance with our cohort of 687 patients which shows a 3.3% increase in risk per point increase on the BDI (supplementary table S5a). Besides the study by McDade one other prospective observational study by Lacson et al. with 6415 patients found similar results when using the 5-item mental health score from the SF-36 questionnaire, where a 1-point increase in depression score was associated with a HR for withdrawal of 1.19 (CI: 1.08–1.31) [11]. However this questionnaire has not been validated for use in the dialysis population for symptoms of depression.

There are also some differences between these previous studies and our present study. First of all, in contradiction to the other studies, we were able to explore the effect of somatic comorbidity on the associations between depressive symptoms and dialysis withdrawal. This is important as anxiety and depression are associated with somatic complaints [10, 31, 32]. Our results indicated however, that somatic comorbidity showed no major changes to the associations between both depression and withdrawal, and anxiety and withdrawal. Second, while McDade had a homogeneous ethnic sample (90% Caucasion). This study replicates these results in a multi-ethnic urban cohort with 49% immigrant patients. Lacson did have a more heterogenous population with 30% non-white patients, however as mentioned above they did not use a depression specific questionnaire that was previously validated in the dialysis population.

Interestingly, our study in a multi-ethnic sample suggests that ethnicity and religion are also associated with dialysis withdrawal. This is not surprising as it is known that certain cultural and religious beliefs are protected factors for end-of-life decisions [33–36]. Future studies are needed to investigate these differences in larger samples.

### Strengths and limitations

This study needs to be interpreted with the following limitations in mind. First, the prospective design of this study is one of the major strengths, however it does limit us in the number of events. Although dialysis withdrawal is one of the main causes of death in this sample, the total number of events is 48, which limited our ability to include a large set of possible confounders in the multivariable models. This could lead to the presence of residual (unmeasured) confounding in our multivariable models. To limit this, we used the Davies comorbidity score which represents multiple somatic comorbidities. Second, in this study withdrawal was defined as the elective cessation of renal replacement therapy without immediate medical indication to do so. This coding, however, does not allow for identifying the underlying reasons for dialysis withdrawal. To be able to investigate the relationship of different factors on the risk of withdrawal from dialysis a clearer and exacter definition is needed to limit heterogeneous results from research. Furthermore, qualitative studies could aid in investigating the decision-making process from a patient and clinician perspective [6].

### Future implications

This study indicates that presence of anxiety and depressive symptoms are not only associated with increased hospitalization rate and increased mortality, but also to dialysis withdrawal, independent of somatic comorbidity. Our findings suggest that screening for symptoms of anxiety and depression may be useful to increase awareness in patients and clinicians about these symptoms and their effect on decision making. We acknowledge that the ability to distinguish demoralization due to somatic burdens from anxiety and depressive disorders remains a challenge. Our study shows that the cut off of common screenings questionnaires may be helpful in understanding the possible risk of these symptoms in relation to dialysis withdrawal. Also, it can be useful to discuss possible treatment options and to make an informed decision on withdrawal. Future studies should further investigate the effect of mental health on end-of-life decisions in dialysis patients, and the role of other patient characteristics like ethnicity and religion, in large multi-ethnic samples. This will help to develop clinical guidelines to improve care for dialysis patients who consider dialysis withdrawal.

## Conclusion

Withdrawal from dialysis therapy is associated with anxiety and depressive symptoms, independent of somatic comorbidities. Further (qualitative) studies are needed to investigate the decision making of patients and clinicians regarding dialysis withdrawal. Increase in knowledge of factors that influence the decisions of dialysis withdrawal could aid patients and clinicians in making an informed decision.

## Electronic supplementary material

Below is the link to the electronic supplementary material.

Supplementary Material 1

## Data Availability

The datasets used and/or analysed during the current study are available from the corresponding author on reasonable request.

## List of Abbreviations

BAI: Beck Anxiety Inventory
BDI-II: Beck’s Depression Inventory, version 2
CCMO: Central Committee on Research Involving Human Subjects
CI: Confidence Interval
DIVERS: Depression-related factors and outcomes In dialysis patients with Various Ethnicities and Races Study
ERA-EDTA: European Renal Association-European Dialysis and Transplant Association
ESKD: End-Stage Kidney Disease
HR: Hazard Ratio
MCS: Mental Component Score
MEC-U: Medical Research Ethics Committees United
PCS: Physical Component Score
QoL: Quality of Life (QoL)
RET: 2012 Guideline External Testing 2012
SF-12: 12-Item Short Form Health Survey
SF-36: 36-Item Short Form Health Survey
