# Supplementary material for: Dialysis withdrawal and symptoms of anxiety and depression: a prospective cohort study
Source: BMC Nephrol. 2023 Jul 24;24:219. doi: 10.1186/s12882-023-03267-2 (PMC10367409; doi:10.1186/s12882-023-03267-2)
Supplement: Supplementary file 1 — Supplementary Material 1 [file 12882_2023_3267_MOESM1_ESM.docx]

Supplementary tables

Supplementary table S1: Rates of dialysis withdrawal stratified by anxiety and depression

| *Incidence rates of dialysis withdrawal per 1000 py* | **Low burden** | **High burden** | **Absolute increase in incidence rate** |
| --- | --- | --- | --- |
| **Anxiety symptoms** | 37.8 / 1000 py | 44.1 / 1000 py | 6.3 / 1000 py |
| **Depressive symptoms** | 27.0 / 1000 py | 40.4 / 1000 py | 13.4 / 1000 py |

Incidence rates are in 1000 person years (py)

For anxiety symptoms a cut-off of BAI≥16 was used.

For depressive symptoms a cut-off of BDI≥13 was used.

Supplementary table S2. The prevalence of dialysis withdrawal stratified by religious groups

| *Baseline self-reported religion* | **Dialysis withdrawal** |
| --- | --- |
| *Religion:*  . Not religious (n220)  . Christian (n213)  . Islamic (n99)  . Hinduism (n44)  . Other religion (n21) | 22 (10%)  15 (7%)  0 (0%)  2 (5%)  0 (0%) |

Supplementary table S3a: Association between depression, anxiety and dialysis withdrawal using education level instead of sex

| *Hazard ratio’s for dialysis withdrawal* | **Model 1**  **(+ age, education, ethnicity)** | **Model 2 (+ comorbidity)** |
| --- | --- | --- |
| **Depressive symptoms** | 2.51 (1.24-5.07) p=0.010 | 2.35 (1.16-4.74) p=0.018 |
| **Anxiety symptoms** | 2.25 (1.06-4.75) p=0.034 | 2.02 (0.95-4.32) p=0.068 |

Model 1 includes both the exposure (anxiety and/or depression) + age, gender and ethnicity.

Model 2 includes both the variables from model 1 + additionally the DAVIES comorbidity score, which includes points for ischemic heart disease, heart failure, peripheral vascular disease, malignancy, diabetes, collagen vascular disease, COPD and others.

Supplementary table S3b: Association between depression, anxiety and dialysis withdrawal using dialysis vintage instead of sex

| *Hazard ratio’s for dialysis withdrawal* | **Model 1**  **(+ age, ethnicity, vintage)** | **Model 2 (+ comorbidity)** |
| --- | --- | --- |
| **Depressive symptoms** | 2.52 (1.25-5.08) p=0.010 | 2.35 (1.16-4.76) p=0.017 |
| **Anxiety symptoms** | 2.33 (1.10-4.92) p=0.027 | 2.03 (0.95-4.35) p=0.067 |

Model 1 includes both the exposure (anxiety and/or depression) + age, gender and ethnicity.

Model 2 includes both the variables from model 1 + additionally the DAVIES comorbidity score, which includes points for ischemic heart disease, heart failure, peripheral vascular disease, malignancy, diabetes, collagen vascular disease, COPD and others.

Supplementary table S4a: Association between depression, anxiety and dialysis withdrawal using the pooled data from a multiple imputed dataset

| *Hazard ratio’s for dialysis withdrawal* | **Crude** | **Model 1**  **(+ age, gender, ethnicity)** | **Model 2 (+ comorbidity)** |
| --- | --- | --- | --- |
| **Depressive symptoms** | 1.57 (0.87-2.83) p=0.133 | 2.05 (1.07-3.94) p=0.031 | 1.95 (1.04-3.77) p=0.046 |
| **Anxiety symptoms** | 1.17 (0.55-2.50) p=0.550 | 1.86 (0.82-4.2) p=0.137 | 1.65 (0.73-3.73) p=0.226 |
| **Both depression and anxiety symptoms** | 1.23 (0.58-2.58) p=0.592 | 1.93 (0.82-4.53) p=0.129 | 1.99 (1.25-3.17) p=0.004 |

Model 1 includes both the exposure (anxiety and/or depression) + age, gender and ethnicity.

Model 2 includes both the variables from model 1 + additionally the DAVIES comorbidity score, which includes points for ischemic heart disease, heart failure, peripheral vascular disease, malignancy, diabetes, collagen vascular disease, COPD and others.

Using a multiple imputation model with 10 repetitions. Indicators include age, sex, ethnicity, married, employed, dialysis modality, diabetes, myocardial infarction, hypertension, malignancy, COPD, self-reported depression, hemoglobin.

Table S4b: Association between depression, anxiety and dialysis withdrawal without patients who received a transplant during follow-up

| *Hazard ratio’s for dialysis withdrawal* | **Crude** | **Model 1**  **(+ age, gender, ethnicity)** | **Model 2 (+ comorbidity)** |
| --- | --- | --- | --- |
| **Depressive symptoms^a^** | 1.62 (0.82-3.20) p=0.168 | 2.53 (1.26-5.10) p=0.009 | 2.43 (1.21-4.93) p=0.013 |
| **Anxiety symptoms^b^** | 1.33 (0.65-2.75) p=0.411 | 2.29 (1.08-4.84) p=0.030 | 2.00 (0.93-4.28) p=0.075 |
| **Both depression and anxiety symptoms^c^** | 1.92 (0.85-4.31) p=0.115 | 3.06 (1.33-7.02) p=0.008 | 2.43 (1.05-5.71) p=0.037 |

Model 1 includes both the exposure + age, gender and ethnicity.

Model 2 includes both the variables from model 1 + additionally the DAVIES comorbidity score, which includes points for ischemic heart disease, heart failure, peripheral vascular disease, malignancy, diabetes, collagen vascular disease, COPD and others.

| ^a^ The presence of depression is defined as a BDI score ≥13 |
| --- |
| ^b^ The presence of anxiety is defined as a BAI score ≥16  ^c^ Based on a grouping variable with both the BDI≥13 AND BAI≥16 versus patients with only anxiety or only depression or none |

**Supplementary table S5a**: Association between depression, anxiety and dialysis withdrawal using continuous variables

| *Hazard ratio’s for dialysis withdrawal* | **Crude** | **Model 1**  **(+ age, gender, ethnicity)** | **Model 2 (+ comorbidity)** |
| --- | --- | --- | --- |
| **Depressive symptoms** | 1.010 (0.975-1.047) p=0.568 | 1.033 (0.994-1.074) p=0.097 | 1.026 (0.987-1.067) p=0.196 |
| **Anxiety symptoms** | 1.012 (0.982-1.043) p=0.442 | 1.034 (1.002-1.066) p=0.038 | 1.026 (0.994-1.059) p=0.112 |

Model 1 includes both the exposure (anxiety and/or depression) + age, gender and ethnicity.

Model 2 includes both the variables from model 1 + additionally the DAVIES comorbidity score, which includes points for ischemic heart disease, heart failure, peripheral vascular disease, malignancy, diabetes, collagen vascular disease, COPD and others.

Supplementary table S5b: Association between health-related quality of life and dialysis withdrawal using continuous variables

| *Hazard ratio’s for dialysis withdrawal* | **Crude** | **Model 1**  **(+ age, gender, ethnicity)** | **Model 2 (+ comorbidity)** |
| --- | --- | --- | --- |
| **Mental component score SF-12** | 0.996 (0.970-1.023) p=0.786 | 0.979 (0.951-1.009) p=0.163 | 0.983 (0.955-1.012) p=0.251 |
| **Physical component score SF-12** | 0.976 (0.950-1.003) p=0.083 | 0.975 (0.948-1.003) p=0.075 | 0.982 (0.955-1.010) p=0.200 |

Model 1 includes both the exposure (anxiety and/or depression) + age, gender and ethnicity.

Model 2 includes both the variables from model 1 + additionally the DAVIES comorbidity score, which includes points for ischemic heart disease, heart failure, peripheral vascular disease, malignancy, diabetes, collagen vascular disease, COPD and others.
